# PLCγ2 impacts microglia-related effectors revealing variants and pathways important in Alzheimer’s disease

**DOI:** 10.3389/fcell.2022.999061

**Published:** 2022-09-06

**Authors:** Ke Li, Beibei Ran, Yu Wang, Lulu Liu, Weidong Li

**Affiliations:** School of Chinese Materia Medica, Beijing University of Chinese Medicine, Beijing, China

**Keywords:** PLCγ2, Alzheimer’s disease, microglia, variant, TREM2

## Abstract

Alzheimer’s disease (AD) is an irreversible neurodegenerative disease mainly characterized by memory loss and cognitive decline. The etiology of AD is complex and remains incompletely understood. In recent years, genome-wide association studies (GWAS) have increasingly highlighted the central role of microglia in AD pathology. As a trans-membrane receptor specifically present on the microglia in the central nervous system, phosphatidylinositol-specific phospholipase C gamma 2 (PLCγ2) plays an important role in neuroinflammation. GWAS data and corresponding pathological research have explored the effects of *PLCG2* variants on amyloid burden and tau pathologies that underline AD. The link between PLCγ2 and other AD-related effectors in human and mouse microglia has also been established, placing PLCγ2 downstream of the triggering receptor expressed on myeloid cells 2 (TREM2), toll-like receptor 4 (TLR4), Bruton’s tyrosine kinase (BTK), and colony-stimulating factor 1 receptor (CSF1R). Because the research on PLCγ2’s role in AD is still in its early stages, few articles have been published, therefore in this paper, we integrate the relevant research published to date, review the structural features, expression patterns, and related pathways of PLCγ2, and summarize the recent studies on important *PLCG2* variants related to AD. Furthermore, the possibility and challenge of using PLCγ2 to develop therapeutic drugs for AD are also discussed.

## Introduction

Alzheimer’s disease (AD) is one of the major diseases associated with aging. It is known for its complex pathophysiology and incurable nature. The increasing number of AD cases has significantly burdened society and patients’ families resulting in AD becoming a serious social and medical problem. By 2050, there are expected to be over 150 million cases of AD worldwide (Rasmussen and Langerman, 2019; Sengoku, 2020). Late-onset AD (LOAD) is the most common type of AD; patients typically present with symptoms after age 65, and the incidence doubles with age every 5 years (Rasmussen and Langerman, 2019; Sengoku, 2020). Of note, statistical studies have estimated the heritability of AD to be between 58% and 79% (Gatz et al., 2006); this high heritability underlies the value of exploring AD treatment modalities from a genetic perspective.

Genome-wide association studies (GWAS) is a novel approach to analyzing the relationship between phenotype and genotype in a population based on genotype information. New research strategies to analyze the genetic variation underlying complex traits have gradually become the mainstream approach to analyzing the heritability of diseases (Dehghan, 2018). While the pathogenesis of LOAD remains inconclusive, over 30 genetic variants have been identified to be associated with LOAD by GWAS; these variants have been or are promising anti-AD targets with therapeutic potential (Karch and Goate, 2015; Efthymiou and Goate, 2017; Bonham et al., 2019; Kunkle et al., 2019; Li et al., 2021). Of note, approximately 40% of the identified genes related to AD are microglia-related (Karch and Goate, 2015), and further analyses of biological functions against these genes have uncovered processes involved in the immune response in the etiology of LOAD. Among these microglia-related genetic factors, most variants were discovered as risk factors for AD progression. In 2017, a rare variant in *PLCG2*, named P522R (rs72824905-G), was reported to be significantly related to LOAD with a protective effect. Since then, phosphatidylinositol-specific phospholipase Cγ2 (PLCγ2) has rapidly become a topic of great interest (Sims et al., 2017).

PLCγ2 is an important signaling protein that, in the brain, is selectively expressed in microglia (Koss et al., 2014; Jackson et al., 2021). Under normal physiological conditions, PLCγ2 exerts its activity through the cleavage of membrane phospholipid phosphatidylinositol-4,5-bisphosphate (PIP2) into diglyceride (DAG) and inositol 1,4,5-triphosphate (IP3) (Berridge, 1995). This process triggers a variety of cell surface receptor signaling pathways that regulate the release of calcium from the endoplasmic reticulum, affecting microglia gene expression, cell viability, and phagocytosis (Koss et al., 2014). The abnormal expression or function of PLCγ2 results in the development of several related pathological manifestations. Some PLCγ2 dysfunction has been demonstrated to be associated with a variety of diseases, including neurodegeneration (Sims et al., 2017; Conway et al., 2018; Dalmasso et al., 2019; van der Lee et al., 2019), immune disorders (Hashimoto et al., 2000; Kim et al., 2004; Regunathan et al., 2006; Afroz et al., 2017; Cheng et al., 2017; Neves et al., 2018; Martin-Nalda et al., 2020), and cancer (Gossmann et al., 2016; Ahn et al., 2017; Jones et al., 2017; Lampson and Brown, 2018; Quinquenel et al., 2019). Jackson and colleagues divided variants of *PLCG2* into three groups. The first group involves inherited mutations on *PLCG2*, which can cause antibody deficiency, immune dysregulation, and, in some cases, autoinflammation. The second group involves acquired mutations on *PLCG2*, which results in constitutive downstream signaling and lymphocyte proliferation, and could be used to treat chronic lymphocytic leukemia. The third group of *PLCG2* variants has effects on neurodegeneration such as AD. The mechanism may involve the promotion of degradation of deleterious neurological aggregates in these diseases (Jackson et al., 2021). According to GWAS and corresponding research *in vitro* and *in vivo*, PLCγ2 might be an important player influencing the formation of Aβ plaques and P-tau, the main hallmarks in AD pathophysiology, and regulating the pathways associated with neuroinflammation and AD-related effector proteins (e.g., TREM2, TLR4, BTK, and CSF1R). Therefore, PLCγ2 is a potential drug target that may be exploited to halt the pathological processes involved in AD. This review aims to comprehensively integrate existing studies of AD-related PLCγ2. In particular, we consider PLCγ2 in microglia with AD-related signaling pathways, in combination with other immune cell functions, and thus describe the possible mechanisms of PLCγ2 in AD. In addition, we also summarize the identified AD-related *PLCG2* variants and the published functional studies of these variants. Finally, this paper also puts forward some existing limitations and future research ideas. We hope this review can provide a timely and practical reference for researchers interested in PLCγ2.

## Structure and expression of phosphatidylinositol-specific phospholipase C gamma 2 in the brain

**FIGURE 1:**
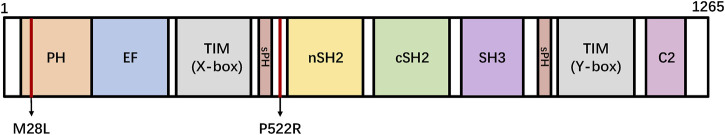
Linear representation of the domains of PLCγ2. *PLCG2* M28L and P522R variants are shown in the domain architecture. N amino-terminus, C carboxyl-terminus, PH: pleckstrin homology domain, EF: EF-hand motif, TIM: TIM barrel, sPH: split PH domain, nSH2: n-terminus Src Homology two domain, cSH2: c-terminus Src Homology two domain, SH3: SRC Homology three domain, C2: C2 domain.

PLCγ2 is one of the 13 members of the phospholipase C (PLC) family (PLC β1-4, PLC γ1-2, PLC δ1-5, and PLC ε1-2), which play various roles in signal transduction pathways (Kadamur and Ross, 2013). Structurally, the catalytic region of PLC consists of the N-terminal pleckstrin homology domain (PH), EF-hand, TIM-like barrel (X-box and Y-box), and C-terminal C2 domain (Magno et al., 2021). Among PLCs, there are two PLCγ proteins, PLCγ1 and PLCγ2, whose unique regulatory domain is inserted between the X box and Y box in the linkers in the TIM-like barrel; It includes a split pleckstrin homology domain (sp), an N-terminal Src homology domain (nSH2), a C-terminal Src homology domain (cSH2) and an SH3 domain (Bunney et al., 2012). (Figure 1). The cSH2 domain of PLCγ2 plays an important role in stabilizing early signal complexes (Wang et al., 2014). The interaction between the cSH2 domain and residues around the active site of the catalyst causes the autoinhibition of PLCγ2 (Milner, 2015). The nSH2 domain is responsible for binding to phosphorylated tyrosine residues in partner proteins such as B-cell linker protein and linker for activation of T cells (Bae et al., 2009; Bunney et al., 2012; Liu et al., 2020). The SH3 domain is highly flexible and can bind to polyproline motifs in adaptors and other proteins (Bae et al., 2017). The PH domain is necessary for PLCγ2 to bind to other proteins on the cell membrane. The instability of this structure may lead to problems in the regulation mechanism of PLCγ2 enzyme activity (Hurley and Grobler, 1997; Falasca et al., 1998; Matsuda et al., 2001).

PLCγ2 is largely considered to be expressed in hematopoietic cells and is involved in the regulation of both the development and function of various hematopoietic cells (Koss et al., 2014). The expression of PLCγ2 in the central nervous system is rarely studied. In the brain, PLCγ2 is mainly expressed in the microglia (Maguire et al., 2021). A recent study has shown that *PLCG2* is a member of a network of co-expressed microglial genes, which is upregulated in brain regions affected by AD pathology, where its upregulation is either resulted from enhanced numbers or activation state of microglia or both (Conway et al., 2018). In addition, gene expression analysis using RNA-Seq data generated from seven brain regions of LOAD participants found that PLCγ2 was over-expressed in the temporal cortex, parahippocampal gyrus, superior temporal gyrus, and inferior prefrontal gyrus. Expression levels of microglia-specific marker genes were significantly associated with PLCγ2 expression levels in the frontal pole, superior temporal gyrus, parahippocampal gyrus, and inferior prefrontal gyrus. Notably, expression levels of PLCγ2 were also shown to correlate significantly with mean amyloid plaque density in the parahippocampal gyrus, superior temporal gyrus, and prefrontal subcortex (Tsai et al., 2022).

## Phosphatidylinositol-specific phospholipase C gamma 2 signaling and its functions

As a downstream enzyme of many signaling proteins, PLCγ2 is actively involved in the maintenance of homeostatic physiological conditions. As mentioned above, PLCγ2 can hydrolyze PIP2 after activation to produce DAG and IP3. PIP2 is located on the cell membrane and can regulate many cellular processes, such as G-protein-coupled receptors, endocytosis, ion channels, and exocytosis (Hammond and Burke, 2020; Katan and Cockcroft, 2020). Another consequence of PIP2 hydrolysis is the production of protons, which cause local changes in the pH of the near-membrane region, thus affecting physiological homeostasis (Huang et al., 2010; Molinari, 2015). Both products of the reaction have roles in intracellular signaling, with IP3 regulating intracellular Ca^2+^ levels by binding to ion channels on the endoplasmic reticulum. DAG remains bound to the membrane and activates protein kinase C (PKC) and Ras guanyl-nucleotide-releasing proteins (RasGRPs), which initiate Nuclear Factor-kappa B (NF-κB) and mitogen-activated protein kinase (MAPK) pathways (Obst et al., 2021).

The function of PLCγ2 in peripheral immune cells has been extensively studied, especially in B cells. Many studies have shown that, as a downstream protein of the B cell receptor (BCR), PLCγ2 is involved in B cell immune responses. PLCγ2 also regulates the function of many other immune cells, by binding to Fc receptors on the membrane of neutrophils, macrophages, mast cells, and NK cells (Wilde and Watson, 2001; Mao et al., 2006; Chiang et al., 2012; Chae et al., 2015; Bae et al., 2017; Zhu et al., 2018). PLCγ2 dysfunction is considered to be a risk factor for a variety of immune-based diseases, including inflammation, allergy, immune deficiency, and even hematologic malignancies (Yu et al., 2005; Abe et al., 2011; Ombrello et al., 2012; Zhou et al., 2012). In the central nervous system (CNS), the expression of PLCγ2 in microglia is more than six-fold higher than in other types of brain cells (Friedman et al., 2018).

Microglia are brain-resident macrophages, and their activation is the initial step in the inflammatory response to CNS diseases. The phenotypic transition of microglia is a dynamic process during the development and progression of AD (Yenari et al., 2010; Chu et al., 2018; Hansen et al., 2018). In the CNS, PLCγ2 is not only expressed in large quantities specifically in microglia but also plays an important regulatory role in the normal function of microglia. Andreone and colleagues have shown that PLCγ2 may affect the survival, proliferation, phagocytosis, and migration of microglia by regulating intracellular calcium release, to help microglia maintain their normal immune response (Andreone et al., 2020). Cellular and animal studies targeting *PLCG2* variants have revealed their impact on microglia under AD conditions, including the association of PLCγ2 with two pathological markers of AD, Aβ and p-tau, and the relationship with many AD-related effectors such as TREM2, TLR4, and others (Shen et al., 2008; Matarin et al., 2015; Sierksma et al., 2020; Tsai et al., 2022).

## Phosphatidylinositol-specific phospholipase C gamma 2 acts as an important player in Alzheimer’s disease pathology

### The pivotal role of phosphatidylinositol-specific phospholipase C gamma 2 in Aβ accumulation

The amyloid hypothesis of AD originates from three proteins encoded by the genes mutated in the autosomal dominant inheritance of AD, namely PSEN1, PSEN2, or APP, which are involved in the metabolism of brain Aβ. A subsequent body of evidence holds that the major pathophysiological events of AD, in chronological order, begin with the deposition of amyloid in plaques, followed by the aggregation of hyperphosphorylated tau into tangles, leading to neurodegenerative changes, and eventually cognitive impairment. For over 30 years, the amyloid hypothesis has been the dominant model in the pathogenesis of AD and has guided drug development approaches (Cirrito et al., 2005; Gandy, 2005; Matarin et al., 2015). Following the discovery of the AD-protective variant of *PLCG2*, the importance of amyloid for the development of LOAD has recently been emphasized. Maguire and colleagues have shown that the *PLCG2* variant P522R, increases the clearance of Aβ-oligomer, by using primary mouse microglia and macrophages (Maguire et al., 2021). The relationship between amyloid plaques and PLCγ2 expression has been supported by increased levels of PLCγ2 throughout disease progression in well-studied models of amyloid pathology, 5x, FAD, TgCRND8, and App NL-G-F/NL-G-F mice (Castillo et al., 2017; Magno et al., 2019). Consistent with these results, as we mentioned above, increased PLCγ2 expression in the brain tissue of LOAD patients was positively correlated with brain amyloid plaque density (Tsai et al., 2022). In addition, the association between PLCγ2 and Aβ aggregation levels was also demonstrated in patients with mild cognitive impairment (MCI) (Kleineidam et al., 2020). All this evidence from cells to humans strongly suggests that PLCγ2 is playing a role in the Aβ pathology.

During AD, the aggregation of Aβ can induce mitochondria apoptosis, disturb the normal metabolic process of cells, inhibit angiogenesis, and induce the release of harmful active compounds (Watson et al., 2005; Holtzman et al., 2011; Landreth et al., 2013). Since PLCγ2 is associated with Aβ and can play a positive regulatory role, we can exploit it as a therapeutic target. Even if the disease cannot be completely eradicated, it may play a role in inhibiting the damage caused by Aβ. However, the present studies on PLCγ2 and Aβ are only limited to characterization, and their complex mechanisms of action require further investigation.

### Phosphatidylinositol-specific phospholipase C gamma 2 modulates tau pathology

Tau is a microtubule-associated protein in the CNS that promotes nucleation, elongation, stabilization, and fasciculation of axonal microtubules and is essential for the assembly of the neuronal cytoskeleton (Ikeda et al., 2010; Sato et al., 2018; Ramirez et al., 2022). In addition to being involved in the assembly of the cytoskeleton, Tau can also bind to other proteins and play a role in cell signaling through phosphorylation. When the phosphorylated regulatory system of Tau is disrupted, such as by hyperphosphorylation, neurological symptoms such as cognitive decline can emerge (Gratuze et al., 2018; Li et al., 2019; Carlomagno et al., 2021). Numerous studies have shown that neuronal tangle formed by hyperphosphorylated polymerization of Tau protein is another hallmark pathological feature of AD in addition to Aβ deposition (Hanger et al., 2007; Reynolds et al., 2008). Recent studies have found a significant correlation between Tau and PLCγ2. A study conducted by Kleineidam and colleagues found that Tau can bind to the SH3 domain of PLCγ2 and that P522R, a protective variant of *PLCG2*, can reduce p-Tau levels in cerebrospinal fluid and delay cognitive decline in individuals with MCI. Notably, MCI patients who carried the P522R variant showed a slower rate of cognitive decline compared to non-carriers, and this effect was mediated by lower P-tau levels in CSF. The effect size of the association of P522R with the cognitive decline and P-tau was similar to that of APOE-ε4, the strongest genetic risk factor for AD (Kleineidam et al., 2020).

## Phosphatidylinositol-specific phospholipase C gamma 2 is the main effector of downstream Alzheimer’s disease-related receptors

### Phosphatidylinositol-specific phospholipase C gamma 2 regulates microglial functions *via* triggering receptor expressed on myeloid cells 2-dependent signaling

During an immune response, microglia are generally and somewhat simplistically considered to differentiate into two phenotypes: the M1 phenotype that causes tissue damage and the M2 phenotype that promotes tissue repair. Phenotypic transition is a dynamic process that plays an important role in neuroinflammation. Under AD conditions, microglia transition from homeostasis to a disease-activated state. The important function of this state is to clear Aβ aggregates by phagocytosis, but unrestrained activation can lead to abnormal chronic inflammation, and cause neurotoxicity. TREM2 is a microglia type I transmembrane protein that is a member of the immunoglobulin superfamily (Paloneva et al., 2002; Yeh et al., 2017; Kulkarni et al., 2021) and has been identified as an immune-associated receptor with high AD risk gene expression (Guerreiro et al., 2013; Slattery et al., 2014). Functional TREM2 may act as an important player in the transition of microglia from homeostatic to pathological, which potentially confers neuroprotection by enhancing microglial function, including an up-regulation of genes implicated in migration, phagocytosis, survival, and lipid metabolism (Krasemann et al., 2017; Qin et al., 2021). TREM2 binds lipids exposed on the surface of apoptotic cells, as well as Aβ, through its ectodomain to activate intercellular signaling pathways, which control innate immune responses (Kleinberger et al., 2014; Jendresen et al., 2017; Lessard et al., 2018; Yang et al., 2019). Human genetic data has exemplified the contribution of TREM2 to the pathology of AD by the identification of rare variants of TREM2 that increase the occurrence of AD by 2∼4-fold (Guerreiro et al., 2013; Slattery et al., 2014). Thus, TREM2 is considered to be a potential biomarker of AD.

**FIGURE 2:**
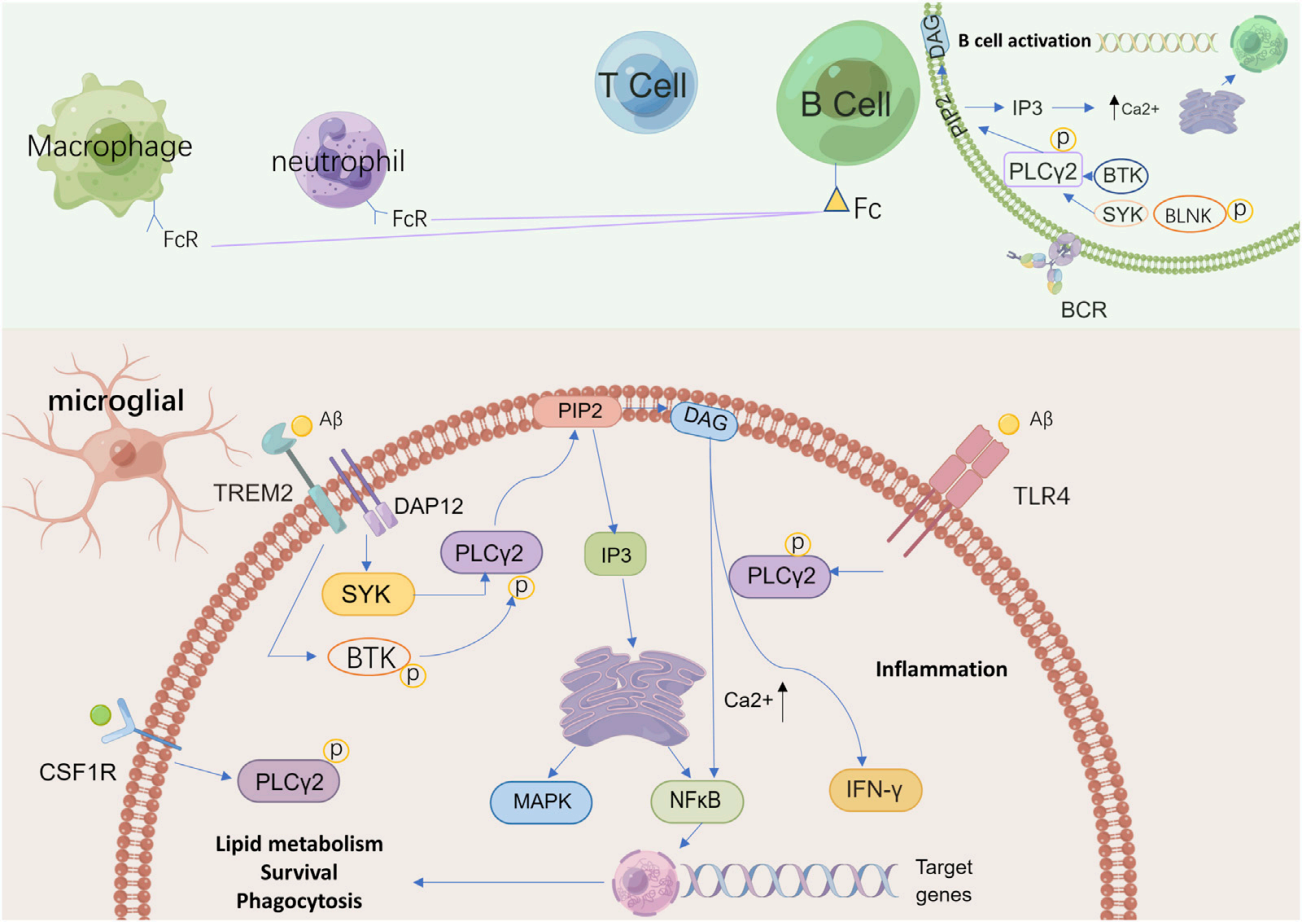
A schematic view of possible pathways related to PLCγ2 in microglia and other immune cells. PLCγ2 belongs to a group of intracellular enzymes that cleave the membrane phospholipid PIP2 to DAG and IP3, resulting in increased calcium signaling. In absence of activating signals, PLCγ2 is neither recruited to the membrane or activated but instead maintained in its autoinhibited form in the cytoplasm. In the brain, PLCγ2 is predominantly expressed by microglia. In microglia, PLCγ2 can be activated downstream of TREM2–DAP12 *via* interaction with SYK to promote beneficial microglial functionality, while also signaling downstream of TLR4 signaling upon ligand stimulation to induce an inflammatory response. The activation of PLCγ2 finally supports lipid metabolism, phagocytosis, and survival. PLCγ2 activation also leads to BCR signaling, including activation of SYK, BTK, and BLNK with increased calcium signaling. This process affects B cell development and increases the production of neutrophils and macrophages. In these immune cells, as a downstream protein in Fc receptor signaling, PLCγ2 regulates calcium-dependent functions to support immune and inflammatory responses. PIP2: phosphatidylinositol-4,5-bisphosphate; DAG: diacylglycerol; IP3: inositol 1,4,5 trisphosphate; TREM2: triggering receptor expressed on myeloid cells two; DAP12: DNAX-activating protein of 12 kDa; SYK: spleen tyrosine kinase; TLR4: toll-like receptor four; PLCγ2: phospholipase C gamma two; BCR: B cell receptor; BTK: Bruton’s tyrosine kinase; BLNK: B cell linker protein.

As a newly discovered AD risk-associated protein and one of the downstream proteins of TREM2, there has been great interest surrounding the role that PLCγ2 plays in the regulation of microglial function by TREM2 and the relationship between PLCγ2 and TREM2 (Figure 2). Andreone and colleagues sought to elucidate the intersection of TREM2 and PLCγ2 signaling and functional expression in human microglia by using human induced pluripotent stem cell (hiPSC) lines that are capable of genetic modification, the results showed that the activation of PLCγ2 was necessary for TREM2 to function normally. Specifically, the effects of TREM2 on microglia survival, proliferation, and phagocytosis are mediated by PLCγ2 signaling (Andreone et al., 2020). Further analysis of the correlation and mode of communication between TREM2 and PLCγ2 by Obst and colleagues revealed that PLCγ2 is a key signaling node linking TREM2 activation with microglia lipid metabolism, furthermore, it was found that the activation of PLCγ2 induced by TREM2 required the recruitment and activation of Syk (Obst et al., 2021). Existing research has not yet clearly elucidated the mechanisms of the TREM2/PLCγ2 pathway; it is suggested that the positive regulation of this pathway may help clear dead cells and pathogenic aggregates by microglia in the course of AD, and thus alleviate the symptoms of AD.

### Phosphatidylinositol-specific phospholipase C gamma 2 is required for toll-like receptor 4 activation

During the pathological progression of AD, the accumulation of Aβ and p-Tau protein can induce apoptosis and increase the expression of inflammatory factors, which can lead to neuroinflammation and nerve injury (Mun et al., 2016). In recent years, inflammatory signaling has become a prominent pathological indicator in AD. These inflammatory signals may be mediated in microglia by toll-like receptors (TLRs), which is a class of transmembrane pattern recognition receptors that are a key component of the innate immune system’s first line of defense against pathogens, binding to pathogen-related and tissue damage-related ligands that signal a need for immune responses (Su et al., 2016). Among the 10 identified functional human TLRs (TLRs 1–10), TLR4 is selectively expressed on the microglia surface. As a binding receptor of Aβ, TLR4 is considered to be a significant mediator of the inflammatory pathophysiological process, and one of the key receptors involved in microglial innate immune activity (Bsibsi et al., 2002; Kawasaki and Kawai, 2014; Huang et al., 2017).

Studies have shown that TLR4 immunoreactivity and proinflammatory cytokine expression are higher in the post-mortem brains of AD patients and the brains of APP/PS1 mice (Walter et al., 2007; Jin et al., 2008). Walter and colleagues have found that the release of neurotoxic products contained in the supernatant of lipopolysaccharide (LPS)-stimulated microglia is dependent on TLR4 activation (Walter et al., 2007). TLR4 antagonists can abrogate Aβ-induced microglia hyperactivation and memory impairment in APP/PS1 mice (Balducci et al., 2017). In addition, fibrillar Aβ has been found to interact with TLR4 to activate the classical signaling pathways required for microglial activation (Udan et al., 2008; Capiralla et al., 2012). Zhou and colleagues have provided a comprehensive review of the effects of TLR4 activity on AD pathology (Zhou et al., 2020). They suggest that TLR4 may be involved in the production of proinflammatory cytokines during AD but that it may also play a neuroprotective role.

PLCγ2 is one of the downstream proteins of TLR4. Normal activity of the TLR4/PLCγ2 pathway plays an important role in maintaining the healthy status of microglia, whereas PLCγ2 activity downstream of TLR4 enables microglia to mount a pro-inflammatory response to specific stimuli. (Chiang et al., 2012; Le et al., 2014; Andreone et al., 2020). Abnormal activation of TLR4/PLCγ2 not only induces a pro-inflammatory response of microglia but also may lead to abnormal intracellular lipid metabolism (Andreone et al., 2020). PLCγ2 has been previously associated with LPS-induced TLR4 endocytosis. Zanoni and colleagues used LPS to stimulate the inflammatory response in AD pathology and found that LPS can trigger the activation of TLR4-dependent PLCγ2, thereby catalyzing the hydrolysis of PIP2. PIP2 degradation attenuates TLR4 signaling by promoting the separation of toll-interleukin-1 receptor domain junction proteins from the plasma membrane (Kagan and Medzhitov, 2006; Zanoni et al., 2011). However, Andreone and colleagues have found that in the absence of TREM2, TLR-dependent PLCγ2 signaling may be enhanced by aberrant lipid metabolism, leading to a high inflammatory state (Andreone et al., 2020). This finding is consistent with what has been observed in AD patients, where microglia can be found to be lipid-loaded and pro-inflammatory (Lue et al., 2001; Foley, 2010; Andreone et al., 2020). Thus, PLCγ2 may act to switch microglia functions between beneficial and deleterious outcomes during disease progression.

### Phosphatidylinositol-specific phospholipase C gamma 2 is required for colony-stimulating factor 1 receptor function

Colony stimulating factor 1(CSF1) is a secreted cytokine that plays a key role in the growth and differentiation of macrophage lineages such as macrophages, osteoclasts, and microglia) (Easley-Neal et al., 2019; Hu et al., 2020; Wu et al., 2020). In the central nervous system, CSF1 is expressed on neurons, microglia, astrocytes, and oligodendrocytes (Chitu et al., 2016; Wylot et al., 2019). CSF1 receptor (CSF1R) is a unique surface receptor that regulates the function of CSF1. Most of these receptors are expressed in microglia, and a few are distributed on neurons in the hippocampus and cortex (Luo et al., 2013; Elmore et al., 2014; Stanley and Chitu, 2014; Xu et al., 2015). In APP/PS1 mice, microglia proliferation is associated with CSF1R-dependent promitosis as AD progresses, and targeted regulation of CSF1R has been shown to improve memory (Olmos-Alonso et al., 2016). Sosna and colleagues found that microglia-mediated CSF1R signaling directly affects amyloid accumulation and neuropathic plaques in neurons (Sosna et al., 2018).

As a downstream signaling protein of CSF1R, PLCγ2 is an integral part of CSF1-induced microglial differentiation after an immune response is triggered (Obba et al., 2015; Magno et al., 2019). Hu and colleagues found that regulation of the CSF1R/PLCγ2/PKCε/CREB signaling pathway reduced microglial proliferation and neuron loss (Hu et al., 2020). As mentioned above, PLCγ2 phosphorylation results in hydrolysis of PIP2 to DAG and IP3. This process leads to phosphorylation of PKCε, which plays an anti-inflammatory role in the CNS by regulating microglia and astrocytes (Gessi et al., 2016; Derouiche and Geiger, 2019). The activation of PKCε further triggers phosphorylation of cyclic adenylate response element binding (CREB) protein, which plays an anti-neuroinflammatory role by inhibiting neuronal apoptosis (Guan et al., 2019; Jaworska et al., 2019). Thus, PLCγ2 is an important downstream signaling protein that depends on the function of CSF1-activated neurons and microglia. However, relevant studies are limited and need further exploration.

### BKT, phosphatidylinositol-specific phospholipase C gamma 2, and microglia

Bruton tyrosine kinase (BTK) is a member of the tyrosine protein kinase subfamily, which is involved in the regulation of peripheral innate immune responses as a key component of B cell receptor signaling (Jumaa et al., 2005). Most of the research on BTK focuses on its regulation of inflammatory response in autoimmune diseases (Yang et al., 2019; Zheng et al., 2021). In recent years, the role of BTK in microglia has received attention, and studies have investigated the role of BTK in AD pathology (Keaney et al., 2019; Weber, 2021). For example, Keaney and colleagues found that BTK can regulate microglia phagocytosis and affect microglia morphology. The inhibition of BTK has been shown to regulate microglial phagocytosis *in vitro* and *ex vivo* (Keaney et al., 2019).

Biochemically, PLCγ2 is downstream of BTK (Watanabe et al., 2001). In addition to the well-established regulation of B cell migration by the BTK/PLCγ2 pathway, *PLCG2* mutations lead to BTK overactivation and a significant increase in intracellular calcium ions, negatively affecting normal B cell development and initiating macrophage and neutrophil production (de Gorter et al., 2007; Zhou et al., 2012). The recruitment of neutrophils by PLCγ2 *via* BTK also modulates E-selectin mediated integrin activation, which causes white blood cells to migrate to inflamed tissues. In terms of microglia function in AD, Keaney and colleagues showed that blocking BTK activity reduced PLCγ2 expression and microglial phagocytosis (Mueller et al., 2010; Keaney et al., 2019). However, our understanding of the regulatory mechanisms of the BTK/PLCγ2 pathway in AD is still limited, and its therapeutic application prospects remain to be further explored.

In addition to participating in the above four signaling pathways closely associated with AD, Claes and colleagues demonstrate the importance of PLCγ2 in the crosstalk between microglia and T cells in the course of AD. Their novel data on P522R variant chimeric AD mice revealed that PLCγ2-dependent microglial activation is involved in promoting the recruitment of T cells and their chemokine expression, and upregulating the expression of multiple antigen-presenting (HLA) genes important to human leukocyte antigen (Claes et al., 2022).

## Phospholipase C-gamma-2 variants in Alzheimer’s disease

### The P522R variant in phospholipase C-gamma-2 shows protection against Alzheimer’s disease

In 2017, the protective and rare coding variant rs72824905 in *PLCG2* (P522R variant) was first reported to be significantly associated with the risk of AD, and this association has been confirmed in GWAS studies in several different populations in subsequent years (Sims et al., 2017; Conway et al., 2018; Dalmasso et al., 2019; Chen et al., 2021; de Rojas et al., 2021). Recently, the P522R variant has become the most studied variant of *PLCG2*. Structurally, the P522R variant causes a proline to arginine substitution leading to an amino change from a non-charged to a positively charged residue. Because proline has one acceptor, and arginine has two acceptors and five hydrogen bonding donor sites, when proline is switched to arginine, hydrogen bonding is increased with an increased number of donors and acceptors. This change may result in a significant change in PLCγ2 activity (Conway et al., 2018; Maguire et al., 2021). In addition, the polymorphism of P522R is located between the nspPH and nSH2 domains, and this change has been shown to enhance PLCγ2 activity in response to stimuli *in vitro* (Everett et al., 2011; Ombrello et al., 2012; Zhou et al., 2012). Computational modeling analysis conducted by Maguire and colleagues suggests that the SH2 domain of the P522R variant is shifted farther to the left and that changes in flexibility and location of this domain may affect the activity of PLCγ2 to catalyze the hydrolysis of PIP2, thereby affecting intracellular Ca^2+^ release (Maguire et al., 2021).

In addition to structural changes, the high functionality of the P522R variant has been confirmed by several studies. By using macrophages and microglia of newly generated P522R-expressing human induced pluripotent cell lines (hiPSC) and knock-in mice, Maguire and colleagues have found that, in all models, cells expressing the P522R mutation show a consistent non-redundant hyper functionality in the context of normal expression of other PLC isoforms, demonstrating that intracellular Ca^2+^ was elevated following PLCγ2 stimulation with an Fc antibody in the P522R variant lines, when compared with the wildtype. Furthermore, expression of the P522R variant leaded to an increased PIP2 depletion and reduced basal PIP2 levels in knock-in mice. This process is related to impaired phagocytosis and enhanced endocytosis (Maguire et al., 2021) (Table 1). Another *in vivo* study by Takalo and colleagues showed that the P522R variant induced a decrease in PIP2 levels to enhance the basic function of PLCγ2, promoting microglia activation without affecting microglia morphology and number (Takalo et al., 2020). Kleineidam and colleagues found that the P522R variant significantly reduced microglia phagocytosis of damaged but surviving neurons and synapses, and reduced pathological manifestations of Tau in the presence of Aβ, thereby reducing cognitive impairment in the course of AD. In addition, the P522R variant can regulate lipid metabolism and restore microglial function by enhancing the clearance of cholesterol esters (Kleineidam et al., 2020).

### The M28L variant in phospholipase C-gamma-2 confers a higher risk for late-onset Alzheimer’s disease

In 2020, again through GWAS analysis, researchers found that a missense variant in *PLCG2*, M28L, was associated with LOAD. In contrast to the P522R variant, which is a protective risk variant for AD, the M28L variant is a deleterious risk variant for AD. The minor allele (T) of M28L in *PLCG2* significantly increased the risk of LOAD [*p* = 0.047, OR = 1.164 (95%CI = 1.002–1.351)] (Kunkle et al., 2019; Tsai et al., 2020). Before this, a large number of studies on the M28L variant have addressed its role in leukemia. These studies mainly used the resistance of the M28L variant to its upstream kinase activator BTK inhibitor to explore the pathogenesis of leukemia (Woyach et al., 2014; Walliser et al., 2016). Methionine at position 28 of the M28L variant is buried in the PH domain at the N-terminal, and this missense mutation may result in less filling of the PH domain and loss of the original stability of the PH domain. The instability in the PH domain has been shown to alter the regulation of protein-protein interactions that have a direct effect on enzyme activity (Tsai et al., 2020). After the GWAS identification of the AD risk effect of the M28L variant, Tsai and colleagues found that M28L variant may induce a dysfunctional microglial phenotype in the course of AD (Tsai et al., 2020). However, the frequency of this mutation is very low, and the interaction between the M28L variant in *PLCG2* and Aβ, Tau, and other pathological markers of AD has not been studied.

Besides these two variants, large meta-analyses have identified three new polymorphisms rs12446759, rs3935877, and rs12444183 that are associated with AD, but with unknown effects on expression, or protein function (de Rojas et al., 2021; Bellenguez et al., 2022).

## Discussion and prospects

Genetic data, protein structure analysis, physiological expression pattern, and pathological function analysis have important guiding roles in clinical diagnosis and treatment. This review comprehensively summarizes the role of PLCγ2 in the progression of AD from the aspects of chemical structure, expression, function, and signaling pathways. Different PLCγ2 signaling pathways may play different regulatory roles in different physiological or pathological conditions. Exploring the potential interactions between PLCγ2 and other receptors can provide effective information for therapeutic strategies for AD. Genetic evidence and corresponding *in vitro* and *in vivo* physiological studies suggest that downstream PLCγ2 signaling, which relies on previously demonstrated AD-associated effectors, plays an important role in the development of the disease. The mechanism mainly involves the activation of the microglial immune response, phagocytosis of the harmful pathological products of AD, and inhibition of neuronal apoptosis and oxidative stress, thereby attenuating cognitive impairment and improving learning ability. We found that, like many important signaling proteins, the role of PLCγ2 in the pathogenesis of AD cannot be exclusively beneficial or harmful. Finding the appropriate activation time and activation level of PLCγ2 is the key to using it as a therapeutic target for AD, and its function in different stages of disease development should be studied more precisely.

The identification of various *PLCG2* variants is critical to deeply exploring the related biological and pathological mechanisms of AD. At the same time, it also provides a new direction for targeted AD treatments and a novel approach to drug development. In addition to the identification of variants, it is essential to further reveal the effects of different variants on protein function and subsequent microglial functional phenotypes in specific disease contexts. Therefore, this review summarizes the current research using different methods and experimental models for the analysis of *PLCG2* P522R variant and attempts to explore the expression characteristics of PLCγ2 in the pathogenesis of AD from the perspective of different risk types of *PLCG2* variants. However, except for the P522R variant, few studies have been conducted on other variants. Therefore, we suggest that researchers should continue to identify AD-related variants and their corresponding biological activities and explore the regulation of different *PLCG2* variants on signal transduction in microglia as part of AD pathophysiology. This will provide critical new information for the understanding and treatment of AD.

Although pieces of evidence have shown that PLCγ2 may be a potential target protein for AD therapy (Magno et al., 2019; Magno et al., 2021; Maguire et al., 2021), the application of these methods in clinical practice is not feasible due to the current lack of effective inhibitors or activators of PLCγ2. In addition, the neurological immune imbalance in AD patients is often particularly complex, and individual differences in drug metabolism can result in up to 70% of patients with AD not responding to a particular drug (Spear et al., 2001). Therefore, a single treatment may not benefit all patients. In recent years, due to the lack of response to drugs or abnormal adverse reactions in some patients, the accidental failure rate of disease treatment has increased. Based on the individual’s genetic information, individualized medicine, which is the best-targeted therapy to prevent or slow down the progression of a disease, has become an effective treatment for many difficult and complicated diseases (Sieber et al., 2014; De Matteis et al., 2018). In combination with other advanced technological tools, such as nanotechnology, which can be used to facilitate drug transport across the blood-brain barrier (BBB) (Saeedi et al., 2019), the application of PLCγ2 to individualized medicine may be a promising direction for AD drug development.

Of note, the *PLCG2* P522R variant is also associated with a reduced risk for dementia subtypes other than AD, such as Lewy body and frontotemporal dementia, and is positively associated with longevity (van der Lee et al., 2019; Chen et al., 2021). Taken together, various findings suggest that modulation of PLCγ2 signaling may be very promising for the development of AD and other neurological diseases. However, the complexity of its function must be considered when designing specific PLCγ2-based strategies for the clinic. Nevertheless, we are confident that PLCγ2 will eventually gain clinical application and that it will become a plausible drug target for the treatment of AD patients in the near future.

**TABLE 1:** Recent research on the P522R variants.

	Experiment model	Main results	References
*In vitro*	Transfected heterologous cell systems (COS7 and HEK293T cells)	1. The P522R variant significantly increases the amount of IP1	Magno et al. (2019)
		2. The P522R variant increases the intracellular Ca^2+^ release	
*In vitro. In vivo*	BMDM derived from Plcγ2-P522R KI mouse model. Mouse BV2 microglia-like cells overexpressing human *PLCG2*-P522R	1. The P522R variant potentiates the primary function of PLCG2 as a PIP2-metabolizing enzyme	Takalo et al. (2020)
		2. The P522R variant directly affects catalysis probably *via* structural changes	
		3. The P522R variant enhances phagocytosis of mouse BV2 microglia-like cells	
		4. The P522R variant moderately increases at least a subset of disease-associated microglia-associated signature genes	
		5. The P522R variant promotes a subset of reactive astrocytes in the KI mouse hippocampus	
		6. The P522R variant exerts both cell-autonomous and non-autonomous effects	
*In vitro. In vivo*	Mouse macrophages, microglia. Kolf2 CRISPR gene-edited *PLCG2*-R522-expressing human-induced pluripotent cell lines (hiPSC). CRISPR gene-edited KI mice	1. The P522R variant enhances the release of cellular calcium ion stores in response to physiologically relevant stimuli like Fc-receptor ligation or exposure to Aβ oligomers	Maguire et al. (2021)
		2. The P522R variant increases stimulus (LPS/Aβ1-42)-dependent PIP2 depletion and reduced basal PIP2 levels	
*In vitro*	*PLCG2*-P522R human iPSC lines and differentiated them into hematopoietic progenitors (HPCs) and microglia (iMGs). Chimeric AD 5XFAD-hCSF1 and WT mice	1. The P522R variant increases HLA-DR expression in plaque-associated xMGs in chimeric AD mice but has no effect on microglial plaque proximity	Claes et al. (2022)
		2. The P522R variant increases the number of xMGs within the cytokine cluster and elevates microglial expression of multiple HLA and chemokine genes in AD chimeric mice	
		3. The P522R variant promotes the expression of antigen presentation genes by xMGs	
		4. Significant increase in the number of CD8^+^ T cells within the brains of P522R KI 5XFAD mice compared to WT 5XFAD mice, with T cells often observed near microglia	
*In vivo*	MIC patients	1. MCI patients who carried the P522R variant shows a slower rate of cognitive decline compared to non-carriers and this effect was mediated by lower pTau181 levels in CSF.	Kleineidam et al. (2020)
		2. The protective effect of P522R was more pronounced in MCI patients with low Aβ1-42 levels	
